# Is cefazolin an appropriate choice for preoperative prophylaxis in patients undergoing surgery for fracture-related infection

**DOI:** 10.1017/ash.2026.10755

**Published:** 2026-07-16

**Authors:** Lauren Daddi, Marjorie Golden, David Frumberg, Anne Spichler-Moffarah

**Affiliations:** Yale University School of Medicine, USA

## Abstract

Prophylaxis against fracture-related infections includes first generation cephalosporins. Open fractures may have soil contamination, increasing risk for infection with gram negative organisms. Broad spectrum antibiotics prevent infections for high-risk patients but cause antibiotic resistance and *C. difficile* colitis. We examined concordance between pathogens and time to infection based on prophylaxis.

## Introduction

Fracture-related infection (FRI) is a serious complication of trauma surgery, causing longer lengths of stay and substantial morbidity. Cefazolin, commonly used for perioperative prophylaxis, may not cover all bacteria causing FRI or prevent polymicrobial infections which occur in up to 45% of patients.^1^ Patients with traumatic fractures have higher risk for infection due to need for multiple surgical procedures including debridement and plastic surgery closure.^2^ Data from Germany showed considerable variation in prophylaxis for fracture fixation surgery.^3^ Surgical Care Improvement Project (SCIP) guidelines^4^ do not distinguish open versus closed fractures and do not select prophylaxis based on preoperative wound cultures. The clinical consensus document published by the American Association for the Surgery of Trauma Critical Care Committee^5^ recommends first generation cephalosporins for prophylaxis when repairing closed extremity fractures and both type I and II open extremity fractures. Guidance published by the American Academy of Orthopedic Surgeons^6^ has similar recommendations but considers broader therapy for type II open fractures. All guidelines recommend broader coverage for type III fractures.^5–7^ This study examined whether antibiotics used for surgical prophylaxis at time of fracture repair provided coverage for organisms subsequently identified in patients with lower extremity FRI, as well as time to development of infection.

## Methods

Retrospective study of adults admitted to Yale-New Haven Hospital (YNHH) from September 2017 to December 2021, with surgical implants placed for lower extremity fracture repair who developed FRI. Patients managed exclusively as outpatients, transferred from other institutions, managed without surgery or with previous FRI were excluded. Subjects identified using ICD 10 codes for hardware infection or prosthetic infection of the lower extremity by YNHH Joint Data Analytics team. Patients taking antibiotics prior to surgery for fracture repair were excluded. FRI definition based on consensus guidelines.^2^

Perioperative prophylaxis was based on SCIP guidelines.^4^ Data was manually extracted by two infectious disease physicians (MPG, ASM) and included demographics, clinical characteristics, choice of prophylaxis, microbiology, and time from fracture repair to development of FRI. Concordance was defined as recovery of a pathogen causing FRI with antibiotic susceptibility to cefazolin. Statistical analysis and box plots using R (4.2.3), Wilcoxon signed-rank tests and χ^2^ test were used to analyze continuous and categorical variables across groups. Gustilo classification system was not recorded as this was a retrospective study, and classification was not consistently documented. Study was approved by Yale School of Medicine Institutional Review Board.

## Results

Patient demographics shown in Table 1. Mean age 51.4 years; 61.4% male. Majority (65.9%) were Caucasian, 18.2% Black and 20.5% Hispanic, with equal distribution between the two groups. Most traumatic fractures (95.5%) were treated with open reduction and internal fixation and rates did not differ between concordant and nonconcordant groups (94.7% vs 96%). MSSA was the commonest pathogen identified, followed by Enterobacterales, MRSA and *P. aeruginosa*. Almost a third (29.5%) had polymicrobial infection (Table 1)

**Table 1.** Clinical characteristics of patients with fracture related infections Table 1 long description.

	Concordance [N=19]	Non-Concordance [N=25]	*P* value^*^
Age (years), median ± SD	57 ± 18.8	48 ± 15.0	0.374
Female Sex (%)	12 (63)	5 (20)	**0.009**
Race			0.435
White (%)	13 (68.4)	16 (64.0)	
Black (%)	2 (10.5)	6 (24.0)	
Not Listed (%)	4 (21.1)	3 (12.0)	
Ethnicity			1.00
Hispanic (%)	4 (21.1)	5 (20.0)	
Non-Hispanic (%)	15 (78.9)	20 (80.0)	
BMI, median ± SD	26 ± 4.93	28 ± 6.96	0.354
Smoking			0.373
Yes	5 (26.3)	11 (44.0)	
No	14 (73.7)	14 (56.0)	
Fracture Location			0.132
Ankle (%)	11 (57.9)	7 (28.0)	
Calcaneus (%)	1 (5.3)	0	
Femur (%)	0	3 (12.0)	
Fibula (%)	0	2 (8.0)	
Tibia (%)	5 (26.3)	11 (44.0)	
Tibia/Fibula (%)	2 (10.5)	2 (8.0)	
Open/Closed Fracture			0.118
Open (%)	6 (31.6)	15 (60.0)	
Closed (%)	13 (68.4)	10 (40.0)	
Polytrauma			0.53
Yes	5 (26.3)	10 (40.0)	
No	14 (73.7)	15 (60.0)	
Fracture Energy			0.511
High	11 (57.9)	18 (72.0)	
Low	8 (42.1)	7 (28.0)	
Unknown	0	0	
Type of Surgery			
ORIF (%)	18 (94.7)	24 (96.0)	1.00
IMN (%)	3 (15.8)	6 (24.0)	0.771
Hardware Status			0.839
Removal	9 (47.4)	10 (40.0)	
Retention	6 (31.6)	10 (40.0)	
Revision	4 (21.1)	5 (20.0)	
Sinus Tract			
Yes			
No	5 (26.3)		
Time Fracture to Infection	14 (73.7)	10 (40.0)	0.530
median ± SD		15 (60.0)	
Time Infection to Surgery	60 ± 99.3		
median ± SD		50 ± 89.7	0.943
Fever	3 ± 27.19		
Yes		4 ± 6.01	0.668
No	4 (21.1)		
Bacteremia	15 (78.9)	2 (8.0)	0.420
Yes		23 (92.0)	
No	1 (5.3)		
Unknown (no BC)	5 (26.3)	1 (4.0)	1.00
Serum WBC, median (IQR)^*^	13 (68.4)	7 (28.0)	
CRP, median (IQR)	8.9 ± 5.4	17 (68.0)	
ESR, median (IQR)		10.6 ± 2.4	
	107 ± 92.8	50 ± 88.1	0.248
	56 ± 28.1	68 ± 36.7	0.366

* only 12/44 pts had this documented.

Of the 44 individuals with FRI, 21 sustained open fractures and 23 had closed fractures. Among patients with open fractures, the majority (85.7%) were high energy fractures. For the 21 patients who sustained open fractures, 31.6% grew bacteria which should have been covered by perioperative prophylaxis (concordant). For the 23 patients who sustained closed fractures, FRI was caused by concordant organisms in 68.4%.

There was a trend toward higher concordance in patients with closed fractures that did not achieve statistical significance. Concordance was seen most commonly (57.9%) in patients who sustained ankle fractures whereas nonconcordance was noted most often (44%) in tibial fractures. Neither achieved statistical significance.

**Figure 1.**
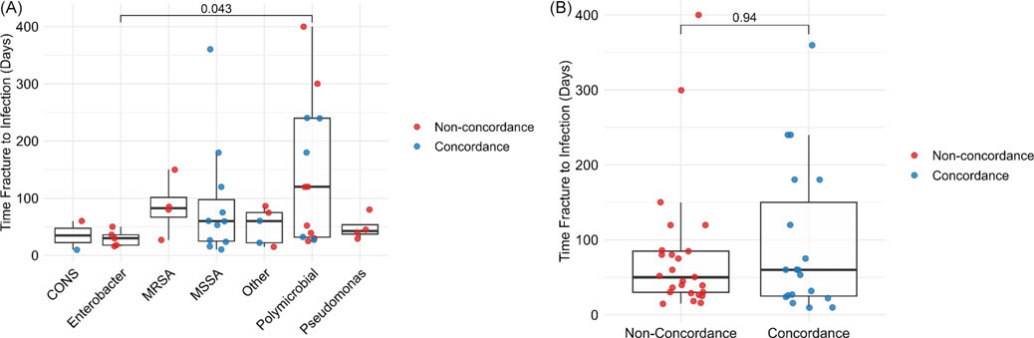
(A) Time from fracture repair to development of FRI by organism. (B) Time from fracture repair to development of FRI based on concordance of antibiotic prophylaxis with organism causing infection.

Of the 44 patients with FRI, 40/44 (90.9%) received prophylaxis with cefazolin alone, 1/44 received cefazolin and gentamicin (2.3%) and 1/44 received clindamycin alone (2.3%). Concordance between prophylactic antibiotic and causative organism in patients who developed FRI was seen in 19/44 (43.2%) and nonconcordance in 25/44 (56.8%). Median time from fracture repair to development of FRI was 60 days (± 99.3 days) in patients with concordant infection and 50 days (± 89.7 days in nonconcordant infections), which was not statistically significant. Figure 1A shows time from fracture repair to development of FRI by organism and Figure 1B shows time from fracture repair to development of FRI based on concordance of antibiotic prophylaxis with infecting organism.

We found no differences in BMI or tobacco use between concordant and nonconcordant patients. Clinical presentation of patients with FRI (fever, leukocytosis, bacteremia, presence of sinus tract and elevated CRP/ESR) were similar in concordant and nonconcordant patients (Table 1).

## Discussion

Cefazolin is considered standard antibiotic prophylaxis for most patients undergoing orthopedic surgery, though questions remain about optimal prophylaxis following fracture repair. Cefazolin’s spectrum of activity does not include MRSA, anaerobes and many gram negative organisms. Fifty percent of organisms causing FRI in our study were not cefazolin susceptible; 22.7% caused by Enterobacterales and *P. aeruginosa.* Over half of patients with FRI (56.8%) showed nonconcordance and this occurred most often in patients with tibial fractures.

Receipt of cefazolin prophylaxis at time of fracture repair did not result in earlier onset of infection with nonconcordant pathogens even in those infected with Enterobacterales and *P. aeruginosa*. We hypothesized that with a large burden of cefazolin resistant organisms present at the time of fracture repair, infection would occur earlier than if infection were caused by cefazolin susceptible pathogens. We were unable to assess adequacy of debridement, and it is possible that aggressive surgical debridement may have prevented early infection. In addition, timing of debridement following injury may be an important risk factor for development of FRI, with studies showing a relationship between timing of debridement and risk for infection following open fracture.^7,8^ Our data extraction excluded patients receiving antibiotics, but we did not capture the specific indications for antibiotic therapy. This may have biased our results with potentially high risk patients already receiving more broad spectrum antibiotics. Another limitation was the small number of patients in our study. Power calculation for our analysis of the relationship between concordance and time from fracture to infection found a small effect size (d = 0.0125) and very low power (5.1%).

Patients undergoing surgery for treatment of closed lower extremity fractures should receive perioperative antibiotics within 1 hour of incision^3^ and we did not find instances of inappropriate timing of prophylaxis. Although we were unable to consistently identify documentation of Gustilo fracture types, only one patient received broad gram-negative coverage (gentamicin) despite 18 patients sustaining high energy open fractures, leading us to speculate that many patients with type III fractures only received cefazolin prophylaxis.

Similar to findings by Depypere et al,^9^ *S aureus* was the most common pathogen isolated with 73% methicillin susceptible.

Cefazolin is widely used for prophylaxis because of its safety, efficacy and tissue penetration. At a time of growing concern about antimicrobial resistance and impacts on the intestinal microbiome, use of more narrow spectrum antibiotics for prophylaxis may minimize collateral damage of antibiotic use. Based on our findings, and weighing risks and benefits of broader therapy, we suggest that routine perioperative prophylaxis with cefazolin appears to be appropriate for patients undergoing fracture repair. There was no difference to time of FRI, even when causative pathogens were resistant to cefazolin. In a time of growing antimicrobial resistance, these data may help inform clinical practice.

## Supporting information

10.1017/ash.2026.10755.sm001 Daddi et al. supplementary material 1 Daddi et al. supplementary material

10.1017/ash.2026.10755.sm002 Daddi et al. supplementary material 2 Daddi et al. supplementary material

## Supplementary material

The supplementary material for this article can be found at https://doi.org/10.1017/ash.2026.10755.

## Table 1 Long description

The table compares patient demographics and clinical characteristics between concordant and non-concordant groups. It has 25 rows and 10 columns. Column headers are Concordeance (N equals 19), Non-Concordeance (N equals 25), and P value. Row labels are Age (years), median plus or minus SD; Female Sex (percent); Race; Ethnicity; B M I, median plus or minus SD; Smoking; Fracture Location; Open/Closed Fracture; Polytrauma; Fracture Energy; Type of Surgery; Hardware Status; Sinus Tract; Time Fracture to Infection; Time Infection to Surgery; Fever; Bacteremia; Serum W B C, median (I Q R); C R P, median (I Q R); and E S R, median (I Q R). Row 1: Concordeance (N equals 19), 57 plus or minus 18.8; Non-Concordeance (N equals 25), 48 plus or minus 15.0; P value, 0.374. Row 2: Concordeance (N equals 19), 12 (63); Non-Concordeance (N equals 25), 5 (20); P value, 0.009. Row 3: Concordeance (N equals 19), White (68.4), Black (10.5), Not Listed (21.1); Non-Concordeance (N equals 25), White (64.0), Black (24.0), Not Listed (12.0); P value, 0.435. Row 4: Concordeance (N equals 19), Hispanic (21.1), Non-Hispanic (78.9); Non-Concordeance (N equals 25), Hispanic (20.0), Non-Hispanic (80.0); P value, 1.00. Row 5: Concordeance (N equals 19), 26 plus or minus 4.93; Non-Concordeance (N equals 25), 28 plus or minus 6.96; P value, 0.354. Row 6: Concordeance (N equals 19), Yes (26.3), No (73.7); Non-Concordeance (N equals 25), Yes (44.0), No (56.0); P value, 0.132. Row 7: Concordeance (N equals 19), Ankle (57.9), Calcaneus (5.3), Femur (0), Fibula (26.3), Tibia (10.5), Tibia/Fibula (0); Non-Concordeance (N equals 25), Ankle (28.0), Calcaneus (0), Femur (12.0), Fibula (44.0), Tibia (8.0), Tibia/Fibula (8.0); P value, 0.132. Row 8: Concordeance (N equals 19), Open (31.6), Closed (68.4); Non-Concordeance (N equals 25), Open (60.0), Closed (40.0); P value, 0.118. Row 9: Concordeance (N equals 19), Yes (26.3), No (73.7); Non-Concordeance (N equals 25), Yes (40.0), No (60.0); P value, 0.53. Row 10: Concordeance (N equals 19), High (57.9), Low (42.1), Unknown (0); Non-Concordeance (N equals 25), High (72.0), Low (28.0), Unknown (0); P value, 0.511. Row 11: Concordeance (N equals 19), O R I F (94.7), I M N (15.8); Non-Concordeance (N equals 25), O R I F (96.0), I M N (24.0); P value, 1.00. Row 12: Concordeance (N equals 19), Removal (47.4), Retention (31.6), Revision (21.1); Non-Concordeance (N equals 25), Removal (40.0), Retention (40.0), Revision (20.0); P value, 0.839. Row 13: Concordeance (N equals 19), Yes (26.3), No (73.7); Non-Concordeance (N equals 25), Yes (40.0), No (60.0); P value, 0.530. Row 14: Concordeance (N equals 19), 60 plus or minus 99.3; Non-Concordeance (N equals 25), 50 plus or minus 89.7; P value, 0.943. Row 15: Concordeance (N equals 19), 3 plus or minus 27.19; Non-Concordeance (N equals 25), 4 plus or minus 6.01; P value, 0.668. Row 16: Concordeance (N equals 19), Yes (78.9), No (5.3), Unknown (no B C) (15.8); Non-Concordeance (N equals 25), Yes (92.0), No (8.0), Unknown (no B C) (0); P value, 0.420. Row 17: Concordeance (N equals 19), 13 (68.4); Non-Concordeance (N equals 25), 7 (28.0); P value, 1.00. Row 18: Concordeance (N equals 19), 8.9 plus or minus 5.4; Non-Concordeance (N equals 25), 17 (68.0); P value, 0.248. Row 19: Concordeance (N equals 19), 107 plus or minus 92.8; Non-Concordeance (N equals 25), 50 plus or minus 88.1; P value, 0.366. Row 20: Concordeance (N equals 19), 56 plus or minus 28.1; Non-Concordeance (N equals 25), 68 plus or minus 36.7; P value, 0.366.

Navigate back to Table 1.
